# Measuring Haitian children's exposure to chikungunya, dengue and malaria

**DOI:** 10.2471/BLT.16.173252

**Published:** 2016-08-31

**Authors:** Mathieu JP Poirier, Delynn M Moss, Karla R Feeser, Thomas G Streit, Gwong-Jen J Chang, Matthew Whitney, Brandy J Russell, Barbara W Johnson, Alison J Basile, Christin H Goodman, Amanda K Barry, Patrick J Lammie

**Affiliations:** a University of Notre Dame Haiti Program, Hôpital Sainte Croix, Rue D’ Accenil No.1, Léogâne, Haiti.; b Centers for Disease Control and Prevention, Atlanta, United States of America (USA).; c Centers for Disease Control and Prevention, Fort Collins, USA.

## Abstract

**Objective:**

To differentiate exposure to the newly introduced chikungunya virus from exposure to endemic dengue virus and other pathogens in Haiti.

**Methods:**

We used a multiplex bead assay to detect immunoglobulin G (IgG) responses to a recombinant chikungunya virus antigen, two dengue virus-like particles and three recombinant *Plasmodium falciparum* antigens. Most (217) of the blood samples investigated were collected longitudinally, from each of 61 children, between 2011 and 2014 but another 127 were collected from a cross-sectional sample of children in 2014.

**Findings:**

Of the samples from the longitudinal cohort, none of the 153 collected between 2011 and 2013 but 78.7% (48/61) of those collected in 2014 were positive for IgG responses to the chikungunya virus antigen. In the cross-sectional sample, such responses were detected in 96 (75.6%) of the children and occurred at similar prevalence across all age groups. In the same sample, responses to malarial antigen were only detected in eight children (6.3%) but the prevalence of IgG responses to dengue virus antigens was 60.6% (77/127) overall and increased steadily with age. Spatial analysis indicated that the prevalence of IgG responses to the chikungunya virus and one of the dengue virus-like particles decreased as the sampling site moved away from the city of Léogâne and towards the ocean.

**Conclusion:**

Serological evidence indicates that there had been a rapid and intense dissemination of chikungunya virus in Haiti. The multiplex bead assay appears to be an appropriate serological platform to monitor the seroprevalence of multiple pathogens simultaneously.

## Résumé

**Objectif:**

Différentier l'exposition au virus chikungunya, récemment introduit, de l'exposition au virus de la dengue et à d'autres pathogènes endémiques en Haïti.

**Méthodes:**

Nous avons procédé à une analyse multiplex à l'aide de billes pour détecter les réponses de l'immunoglobuline G (IgG) à un antigène recombinant du virus chikungunya, à deux particules pseudo-virales de la dengue et à trois antigènes recombinants de *Plasmodium falciparum*. La plupart des échantillons de sang (217) analysés ont été prélevés de façon longitudinale sur chacun des 61 enfants, entre 2011 et 2014, et 127 autres ont été prélevés sur un échantillon transversal d'enfants en 2014.

**Résultats:**

Dans la cohorte longitudinale, aucun des 153 échantillons prélevés entre 2011 et 2013 n'affichait de réponse positive de l'IgG à l'antigène du virus chikungunya, mais 78,7% (48/61) de ceux prélevés en 2014 étaient en revanche positifs. Dans l'échantillon transversal, des réponses positives ont été relevées chez 96 enfants (75,6%), avec une prévalence similaire dans tous les groupes d'âge. Sur ce même échantillon, des réponses à l'antigène du paludisme n'ont été détectées que chez huit enfants (6,3%) mais la prévalence globale des réponses de l'IgG aux antigènes du virus de la dengue était de 60,6% (77/127) et augmentait progressivement avec l'âge. L'analyse géographique a révélé que la prévalence des réponses de l'IgG au virus du chikungunya et à l'une des particules pseudo-virales de la dengue diminuait à mesure que le site d'échantillonnage s'éloignait de la ville de Léogâne en direction de l'océan.

**Conclusion:**

Les preuves sérologiques indiquent que la propagation du virus du chikungunya en Haïti a été rapide et intense. L'analyse multiplex à l'aide de billes s'est avérée être une plate-forme sérologique appropriée pour contrôler simultanément la séroprévalence de plusieurs pathogènes.

## Resumen

**Objetivo:**

Diferenciar la exposición al nuevo virus chikungunya de la exposición al virus endémico del dengue y a otros patógenos en Haití.

**Métodos:**

Se utilizó un ensayo multiplex de microesferas para detectar las respuestas de la inmunoglobulina G (IgG) ante un antígeno recombinante del virus chikungunya, dos partículas similares al virus del dengue y tres antígenos recombinantes de *Plasmodium falciparum*. La mayoría (217) de las muestras de sangre investigadas se recogieron de forma longitudinal, de cada uno de los 61 niños, entre 2011 y 2014, pero en 2014 también se recogieron otras 127 de una muestra transversal de niños.

**Resultados:**

De las muestras de la cohorte longitudinal, ninguna de las 153 muestras recogidas entre 2011 y 2013 dio un resultado positivo de respuestas de la IgG al antígeno del virus chikungunya, pero sí que lo dieron el 78,7% (48/61) de las muestras recogidas en 2014. En la muestra transversal, se detectaron dichas respuestas en 96 (75,6%) de los niños y se mostró una prevalencia similar en todos los grupos de edades. En la misma muestra, únicamente se detectaron respuestas al antígeno de la malaria en ocho niños (6,3%), aunque la prevalencia de las respuestas de la IgG a los antígenos del virus del dengue fue del 60,6% (77/127) en general y aumentaba de forma estable con la edad. Los análisis territoriales indicaron que la prevalencia de las respuestas de la IgG al virus chikungunya y a una de las partículas similares al virus del dengue se redujo conforme el lugar de la muestra se alejaba de la ciudad de Léogâne y se acercaba al océano.

**Conclusión:**

La prueba serológica indica que se ha producido una diseminación rápida e intensa del virus chikungunya en Haití. Parece que el ensayo multiplex de microesferas es una plataforma serológica adecuada para supervisar la seroprevalencia de varios patógenos al mismo tiempo.

## ملخص

**الغرض:**

التمييز بين معدلات التعرض لفيروس شِيكُونْغُونْيا الذي تم الكشف عنه مؤخرًا ومعدلات التعرض لفيروس الضنك المتوطّن والعوامل الأخرى المسببة للأمراض في دولة هايتي.

**الطريقة:**

استخدمنا المقايسة المتعددة للحبيبات لاكتشاف أية استجابات من جانب الغلوبولين المناعي G (IgG) إلى مستضد مؤتلف لفيروس شِيكُونْغُونْيا، وجسيمين مشابهين لفيروس الضنك، وثلاثة مستضدات مؤتلفة لطُفيل المتصورة المنجلية. لقد تم جمع معظم عينات الدم (البالغ عددها 217) التي خضعت للاستقصاء من كل 61 طفلًا على مدى فترة طويلة تمتد بين عامي 2011 و2014، ولكن في عام 2014، تم جمع 127 عينة أخرى لقطاعات متعددة من الأطفال.

**النتائج:**

لم تُظهر أي من العينات المُجمّعة فيما بين عامي 2011 و2013 والبالغ عددها 153 نتائج إيجابية عن وجود استجابات من جانب IgG إلى المستضد المؤتلف لفيروس شِيكُونْغُونْيا باستثناء ما يعادل 78.7‏% (‏48/61) من العينات التي تم جمعها في عام 2014 وذلك من أصل العينات المستخرجة من مجموعة الأطفال على مدى فترة طويلة. في العينة المستخرجة من عدة قطاعات، تم اكتشاف تلك الاستجابات في 96 طفلاً (فيما يعادل 75.6%) وكان معدل انتشارها متشابهًا في جميع الفئات العمرية. وفي نفس تلك العينة، لم يتم اكتشاف أية استجابات لمستضد الملاريا سوى في ثمانية أطفال (فيما يعادل 6.3‏%) بينما بلغ إجمالي معدل انتشار الاستجابات من جانب IgG إلى مستضدات فيروس الضنك 60.6‏% (‏77/127) واستمر في الازدياد بثبات مع ازدياد العمر. وأوضح التحليل المكاني أن معدل انتشار الاستجابات من جانب lgG إلى فيروس شِيكُونْغُونْيا وأحد الجسيمين المشابهين لفيروس الضنك قد ازداد بتغيير موقع أخذ العينات من مدينة يوغان انتقالاً باتجاه المحيط.

**الاستنتاج:**

تشير الأدلة المصلية إلى انتشار فيروس شِيكُونْغُونْيا بسرعة وبشدّة في دولة هايتي. كما تُظهر المقايسة المتعددة للحبيبات أن بإمكانها أن تصبح نظامًا مصليًا مناسبًا للإشراف على معدل الانتشار المصلي لعدة عوامل مسببة للأمراض في نفس الوقت.

## 摘要

**目的:**

旨在将海地地区接触新流入的基孔肯雅病毒与接触地方性登革病毒以及其他病原体进行区分。

**方法:**

我们采用多重微球试验以检测免疫球蛋白 G (IgG) 对重组基孔肯雅病毒抗原、两种类似登革病毒的微粒和三种重组*恶性疟疾*抗原的反应。 研究中的大部分血样（217 例）是采用纵向方式在 2011 年到 2014 年自 61 位儿童采集而来，但还有 127 例是采用横断面儿童采样的方式于 2014 年采集而来。

**结果:**

在纵向群组样本中，2011 年到 2013 年间采集的 153 例样本中没有一例在 lgG 对基孔肯雅病毒抗原有反应中显示阳性，但在 2014 年采集的样本中 78.7% (48/61) 显示阳性。 在横断面样本中，我们发现其中 96 位儿童 (75.6%) 存在此类反应，并且在所有年龄段都有相似概率。 在相同样本中，仅在八位儿童 (6.3%) 中检测到对疟疾抗原有反应。但整体而言，IgG 对登革病毒抗原有反应的概率是 60.6% (77/127)，并且随着年龄增长而稳步增长。 空间分析显示，IgG 对基孔肯雅病毒和其中一种类似登革病毒的微粒有反应的概率随着取样地点从莱奥甘转移至海边而有所降低。

**结论:**

血清学证据表明海地地区的基孔肯亚病毒已经快速密集传播。 多重微球试验似乎是适当的血清学平台，可同时监控多种病原体血清阳性率。

## Резюме

**Цель:**

Провести различия между подверженностью воздействию недавно интродуцированного вируса чикунгуньи и воздействию эндемического вируса лихорадки денге и других болезнетворных микроорганизмов в Гаити.

**Методы:**

С помощью мультиплексного анализа с использованием гранул авторы статьи выявили реакцию иммуноглобулина G (IgG) на антиген рекомбинантного вируса чикунгуньи, две вирусоподобные частицы денге и три антигена рекомбинантного паразита Plasmodium falciparum. Забор большинства (217) исследованных образцов крови совершался в течение нескольких лет у 61 ребенка между 2011 и 2014 годами, а 127 образцов были взяты у детей в рамках профильной пробы в 2014 году.

**Результаты:**

Ни для одного из 153 образцов, отобранных между 2011 и 2013 годами у детей из когорты долгосрочного исследования для анализа на реакцию IgG на антиген вируса чикунгуньи, не был получен положительный результат; однако 78,7% (48/61) образцов, полученных у детей из этой же группы в 2014 году, дали положительный результат. В профильной выборке реакция была обнаружена у 96 (75,6%) детей и наблюдалась со схожей частотностью во всех возрастных группах. В той же выборке реакция на малярийный антиген была обнаружена только у восьми детей (6,3%), однако общая распространенность реакции IgG на антигены вируса денге составила 60,6% (77/127) и с возрастом стабильно увеличивалась. Анализ на распределение по местности показал, что распространенность реакции IgG на вирус чикунгуньи и вирусоподобные частицы денге уменьшалась по мере перемещения места отбора проб от города Леоган в сторону океана.

**Вывод:**

Выявленные серологические реакции свидетельствуют о произошедшем скоротечном и усиленном распространении вируса чикунгуньи в Гаити. Мультиплексный анализ с использованием гранул служит подходящей базой для серодиагностики для одновременного отслеживания распространенности положительных серологических реакций на несколько болезнетворных микроорганизмов.

## Introduction

The symptoms of human infections with dengue virus are often so similar to those of infection with chikungunya virus that clinical differentiation of the two types of infection is difficult. Infection with chikungunya virus, which belongs to the genus *Alphavirus* in the family Togaviridae, can cause conjunctivitis, debilitating polyarthralgia, diarrhoea, headache, fatigue, fever, myalgia, nausea, rashes and vomiting.^1^ Infection with dengue virus, which belong to the genus *Flavivirus* in the family Flaviviridae, is also often associated with severe joint pain and can lead to dengue fever or to the potentially deadly severe dengue. *Aedes aegypti* and *Ae. albopictus* are the main vectors of both chikungunya and dengue virus.

Although the disease we now call chikungunya appears to have been initially described in the 1820s, almost simultaneously in East Africa – the area now known as the United Republic of Tanzania – and India, chikungunya virus was not isolated until 1952.^1^^–^^3^ Human infections with this virus were reported in Bangkok, Thailand, in the 1960s and in India between 1963 and 1973.^4^^–^^6^ Such infections may rapidly develop into large epidemics. In 2006 on an overseas department of France – the island of La Réunion – there was an epidemic where the incidence of chikungunya peaked at more than 40 000 cases per week.^7^ By early 2013, chikungunya had been reported in Africa, Asia, Europe and parts of Oceania.^8^

Since both *Ae. aegypti* and *Ae. albopictus* occur in Haiti, chikungunya virus was expected to arrive in the country and to be disseminated rapidly.^9^^,^^10^ In December 2013, the World Health Organization (WHO) reported the local spread of the virus in nearby Saint Martin – another overseas department of France.^11^ On 6 June 2014, the United States Centers for Disease Control and Prevention (CDC) reported 6318 chikungunya cases in Haiti and, by the end of 2014, transmission of chikungunya virus had been reported throughout the Caribbean basin.^12^^,^^13^

Because the symptoms and epidemiology of chikungunya and dengue fever are similar and occur against a backdrop of other infectious diseases, our objective was to identify and assess immunoglobulin G (IgG) responses to these closely related pathogens. A search, on 6 November 2015, for both “multiplex” and “chikungunya” in the titles and abstracts of the published articles listed by PubMed resulted in a list of 21 articles. Of these articles, nine described laboratory techniques based on the reverse-transcription polymerase chain reaction, two used assays based on the same reaction to identify chikungunya virus in the field^14^^,^^15^ and one described an antibody-neutralization technique.^16^ As multiplex bead assays allows the simultaneous collection of data on antibody responses to multiple antigens,^17^^–^^23^ we investigated the use of such an assay to assess the IgG responses to antigens from chikungunya and dengue virus and *Plasmodium falciparum* in Haitian children. Our post-hoc testing of blood samples was primarily done to generate epidemiological data about the introduction of chikungunya virus in Haiti and was not aimed at diagnosis or case management.

## Methods

### Study population and design

The study protocol was reviewed and approved by the Ethics Committee of the Hôpital Sainte Croix, Léogâne, Haiti, and the Institutional Review Boards of both the CDC, Atlanta, United States of America, and the University of Notre Dame, Notre Dame, USA.

We collected blood spot samples from a longitudinal cohort of 61 children – all residents in the small coastal town of Ca Ira – at three or all four of four time points: December 2011, February 2013, December 2013 and August 2014. All the blood spot samples collected before 2014 had been collected, from children who were aged 2–10 years in December 2011, for filariasis surveillance. In August 2014, specifically for our investigation of the bead assay, we collected blood from all 61 previously sampled children and from another 127 children from Ca Ira, then aged 2–10 years, who had not been sampled before. We considered the 127 children to represent a cross-sectional sample.

Prior to the collection of each blood spot sample, a local community health worker obtained the informed consent from a parent of the sampled child. To prepare each sample, 10 µl whole blood was collected from a finger-prick and transferred to one unused extension of a piece of filter paper with six circular extensions (TropBio Pty Ltd, Townsville, Australia). After collection, the blood spots were allowed to dry and then stored at −20 °C until tested.^18^

### Antigens

We used one recombinant chikungunya virus antigen – that is, mutant A226V envelope 1 antigen (CTK Biotech, San Diego, USA) – two dengue virus antigens – propagated using a eukaryotic plasmid vector that expressed the premembrane/membrane and envelope proteins that self-assemble into two different non-infectious virus-like particles known as DENV-2 and DENV-3^24^^,^^25^ – and three recombinant antigens based on merozoite surface protein 1 of *P. falciparum*.^26^^–^^29^ The DENV-2 virus-like particle has epitopes for antibodies to dengue virus serotypes 2 and 4 whereas the DENV-3 has epitopes for antibodies to serotypes 3 and 1.^30^ The recombinant *P. falciparum* antigens represented a 42-kDa fragment from clone FVO, a 42-kDa fragment from clone 3D7 and a 19-kDa fragment from clone 3D7 (Zentrum für Molekulare Biologie der Universität Heidelberg, Heidelberg, Germany) that was linked with glutathione-*S*-transferase.^26^^–^^29^

### Antigen coupling

1-Ethyl-3-(3-dimethylaminopropyl) carbodiimide (Calbiochem, Woburn, USA) was used to convert the carboxyl groups on carboxylated polystyrene beads (SeroMap Beads; Luminex Corporation, Austin, USA) to esters. The esters on the so-called activated beads could then react with amine groups on the antigens to form covalent amide bonds between the beads and the antigens. We coupled 12.5 million activated beads – in phosphate-buffered saline at pH 7.2 – with 30 µg of each dengue virus-like particle and 7.5 µg of the chikungunya virus antigen. For the malaria antigens and glutathione-*S*-transferase, we coupled 12.5 million activated beads, in 50 mmol/L 2-(*N*-morpholino) ethanesulfonic acid buffer with 0.85% (w/v) NaCl, at pH 5.0 – with 28 µg of the 19-kDa fragment, from clone 3D7, linked to glutathione-*S*-transferase, 15 µg of the 42-kDa fragment from clone 3D7, 15 µg of the 42-kDa fragment from clone FVO, and 12 µg of glutathione-*S*-transferase. Coupling efficiency was evaluated using monoclonal antibodies and reference sera that were known to be highly reactive to the antigens.^31^^,^^32^

### Blood spot elution

Each dried blood spot was eluted overnight, at 6 °C, in 500 µL of a buffer consisting of phosphate-buffered saline, at pH 7.2, containing 0.5% (w/v) bovine serum albumin, 0.5% (w/v) polyvinyl alcohol, 0.8% (w/v) polyvinylpyrrolidone, 0.5% (w/v) casein, 0.3% (w/v) Tween 20 and 0.02% (w/v) sodium azide. To adsorb any antibodies to *Escherichia coli* – that might have reacted with *E. coli* proteins inadvertently coupled to the beads – each elution was diluted 1:4 with the same buffer containing sufficient crude *E. coli* extract to give a final concentration of 3.0 µg extract per mL.^17^ All serum specimens were diluted, 1:400, in the same buffer with the *E. coli* extract.

### Bead assay

#### Procedure

The *E. coli*-treated samples were stored overnight at 6 °C and then clarified by centrifugation at 20 238 *g* for 10 minutes. All incubation steps were conducted at room temperature – i.e. 20–22 °C – in 96-well filter-bottom plates (Merck KGaA, Darmstadt, Germany). For each assay, a 50-µl clarified sample was added to a single well containing 1500 antigen-coupled beads from each coupled-bead classification and incubated, with gentle shaking, for 1.5 hours. The assay was then run as previously described.^17^ Median fluorescence intensities between 1 and 32 766 were evaluated in a plate reader (Luminex Corporation, Austin, USA) equipped with Bio-Plex Manager 6.1 software (Bio-Rad, Hercules, USA). The background fluorescence – from wells containing no primary antibody – was subtracted from the mean of the median fluorescence intensities of duplicate wells to yield background-adjusted median fluorescence intensities. To evaluate inter-plate consistency, positive controls, diluted to yield low to moderate fluorescence intensities, were run on each plate.

#### Positivity thresholds

For setting the threshold for assay positivity for the chikungunya virus antigen, we used assay results for the blood spots collected, before 2014, from the longitudinal cohort of Haitian children. For all the other thresholds, we used the bead assay to test 86 sera from adults who claimed to have never left the United States (Table 1). Any serum with background-adjusted fluorescence more than 3.0 standard deviations above the mean was considered to be an outlier and ignored. For each antigen, the threshold for assay positivity was set as the mean background-adjusted result plus 3.0 standard deviations for the relevant non-outliers.

**Table 1:** Reference values and positivity thresholds for a multiplex bead assay based on antigens from chikungunya and dengue viruses and *Plasmodium falciparum*

Pathogen, antigen	Reference sample**^a^**	Fluorescence intensity**^b^**	
		Median for study samples (range)	Threshold
**Chikungunya virus**			
Envelope 1 antigen of mutant A226V	Blood	101 (−9 to 30 027)	640
**Dengue virus**			
DENV-2 virus-like particle	Sera	10 168 (−18 to 28 395)	982
DENV-3 virus-like particle	Sera	15 047 (3 to 30 214)	3615
***Plasmodium falciparum***			
19-kDa fragment of MSP-1 of clone 3D7, linked to GST	Sera	8 (−6 to 21 627)	105
42-kDa fragment of MSP-1 of clone 3D7	Sera	29 (2 to 23 913)	189
42-kDa fragment of MSP-1 of clone FVO	Sera	20 (0 to 20 489)	79
**GST^c^**	Sera	7 (−4 to 173)	NA

GST: glutathione-*S*-transferase; MSP-1: merozoite surface protein 1; NA: not applicable.

^a^ The reference samples were 86 sera from adults who claimed to have never left the United States or 156 spots of blood collected from 62 Haitian children between 2011 and 2013.

^b^ Each reported intensity is the result of subtracting background fluorescence, from wells containing no primary antibody, from the mean of the median fluorescence intensities of duplicate wells.

^c^ Used as a protein-negative control.

A test blood spot was considered positive for an IgG response to *P. falciparum* if it was found positive for at least two of the three *P. falciparum* antigens we investigated. A test blood spot was considered positive for an IgG response to dengue virus if it was found positive for one or both of the virus-like particles we investigated.

#### Validating assay for chikungunya antigen

We used both the bead assay and an enzyme-linked immunosorbent assay (ELISA) to test 78 sera – i.e. 26 sera collected from Haitian adults in 1996, when chikungunya was known to be present in Cambodia but not in Haiti,^13^ and 52 sera collected from Cambodian adults in 2012 – for IgG that reacted with chikungunya virus antigen. The viral antigen used in the ELISA came from the brain of a suckling mouse and was captured with a monoclonal antibody.^33^ Any IgG from a test serum that reacted with this antigen was probed with goat anti-human IgG linked to alkaline phosphatase. Colour was developed using disodium p-nitrophenyl phosphate and read at 405 nm.^33^

### Spatial analysis

The positions of the 143 households that participated in the blood spot collection in August 2014 were mapped using a global positional system and ArcGIS software (ESRI, Redlands, USA). The same software was then used to perform a so-called hot-spot analysis of the corresponding bead assay results for three of the test antigens: chikungunya virus, the DENV-2 virus-like-particle and the 19-kDa malarial antigen linked to glutathione-*S*-transferase. For this analysis, we used a 400-m zone of indifference to reflect the mean range of an adult female *Ae. aegypti*.^34^ To prevent association of the results with household locations, we aggregated the Z-scores for the Getis–Ord *G*_i_ statistic^35^ and displaced the corresponding symbols from the actual locations.

### Statistics

We used Spearman’s correlation to investigate the levels of association between the IgG responses for the test antigens. A *P*-value of less than 0.05 was considered indicative of statistical significance. Inter-plate consistency was evaluated as the percentage coefficient of variation for the diluted positive controls.

## Results

The positivity thresholds are summarized in Table 1. Inter-plate consistency was good, with the percentage coefficient of variation always less than 10.6. In the bead assay, monoclonal antibodies and sera known to be highly reactive to the antigens gave high background-adjusted fluorescence intensities, indicating antigen integrity and adequate antigen coupling.^31^^,^^32^^,^^36^

### Validation

In the capture ELISA, using chikungunya virus from a mouse, only 50 of the 78 tested sera gave interpretable results.^33^ When the results of this assay were considered to be the so-called gold standard, the bead assay appeared to be 90% sensitive and 85% specific.

### Longitudinal cohort

#### Prevalence of pathogen exposure

Within the longitudinal cohort (Table 2), no evidence of exposure to chikungunya virus was observed from 2011 to 2013 but 78.7% (46/61) of the samples tested in 2014 showed evidence of such exposure. The prevalence of exposure to dengue virus rose from 62.3% (38/61) in December 2011 to 79.0% (27/34) in February 2013 and then remained relatively unchanged for the remainder of the study. The corresponding prevalence of *P. falciparum* never exceeded 12% throughout the study. No exposure to any of the three pathogens investigated was evident in 4.9% (3/61) of the longitudinal cohort.

**Table 2:** Percentages of blood spots from 61 Haitian children found bead-assay-positive for immunoglobulin G responses to antigens representing chikungunya and dengue viruses and *Plasmodium falciparum,* 2011–2014

Pathogen	No. positive (%)			
	December 2011 (*n* = 61)	February 2013 (*n* = 34)	December 2013 (*n* = 61)	August 2014 (*n* = 61)
Chikungunya virus	0 (0)	0 (0)	0 (0)	48 (78.7)
Dengue virus	38 (62.3)	27 (79.4)	49 (80.3)	48 (78.7)
*Plasmodium falciparum*	7 (11.5)	1 (2.9)	5 (8.2)	4 (6.6)

#### Temporal trends

**Fig. 1:**
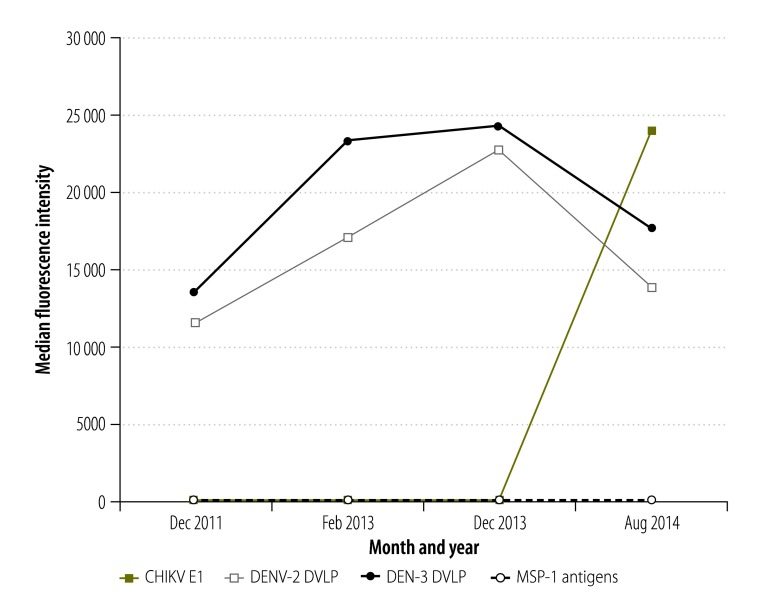
Fluorescence intensities recorded in multiplex bead assays based on antigens representing chikungunya and dengue viruses and *Plasmodium falciparum,* Haiti, 2011–2014

The temporal trends in the background-adjusted fluorescence intensities are summarized in Fig. 1. In the assays based on chikungunya virus antigen, the median intensities were low between 2011 and 2013 but rose sharply in 2014. In contrast, in the assays based on either of the dengue antigens we tested, median intensities were relatively low in 2011 and increased through 2013 but then fell slightly in 2014. In the assays based on any of the malarial antigens, median intensities remained low throughout the study period.

CHIKV E1: chikungunya virus envelope 1; DVLP: dengue virus-like particle; MSP-1: merozoite surface protein 1.

Notes: The assays were used to investigate blood spots from a longitudinal cohort of 61 children. The intensities shown are each the result of subtracting background fluorescence, from wells containing no primary antibody, from the mean of the median fluorescence intensities of duplicate wells. The three *P. falciparum* antigens tested – i.e. 19-kDa, 42-kDa and 42-kDa fractions of the MSP-1 clones 3D7, 3D7 and FVO, respectively, gave almost identical results.

#### Results for one child

**Fig. 2:**
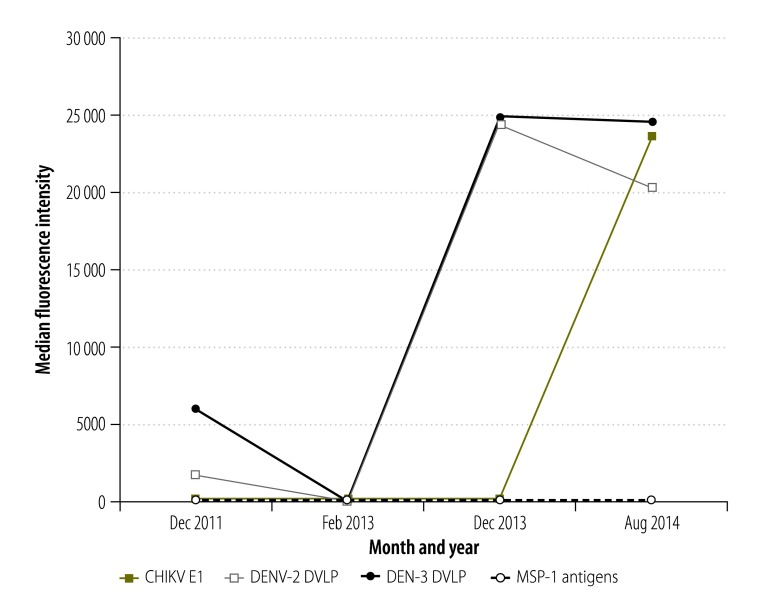
Fluorescence intensities, for one child, recorded in multiplex bead assays based on antigens representing chikungunya and dengue viruses and *Plasmodium falciparum,* Haiti, 2011–2014

The assay results for one child who was sampled at each of the four time points are summarized in Fig. 2. This child was only found to have high IgG responses to the chikungunya virus antigen in 2014. In the bead assays based on the dengue virus-like-particles, this child was found barely to exceed the positivity threshold in December 2011, to be negative in February 2013 and then to be well over the positivity threshold in December 2013 – demonstrating a secondary immune response to the dengue antigens. The child was never found positive in the assays for malarial antigens.

CHIKV E1: chikungunya virus envelope 1; DVLP: dengue virus-like particle; MSP-1: merozoite surface protein 1.

Notes: The intensities shown are each the result of subtracting background fluorescence, from wells containing no primary antibody, from the mean of the median fluorescence intensities of duplicate wells. The three *P. falciparum* antigens tested – i.e. 19-kDa, 42-kDa and 42-kDa fractions of the MSP-1clones 3D7, 3D7 and FVO, respectively, gave almost identical results. In December 2011, the child was aged two years.

### Cross-sectional sample

#### Prevalence of pathogen exposure

Of the 127 children who each provided a single blood spot in 2014, 96 (75.6%), 77 (60.6%) and eight (6.3%) were found seropositive for chikungunya virus, dengue virus and *P. falciparum*, respectively, and 17 (13.4%) showed no evidence of exposure to any of these pathogens.

#### Age-specific trends

**Fig. 3:**
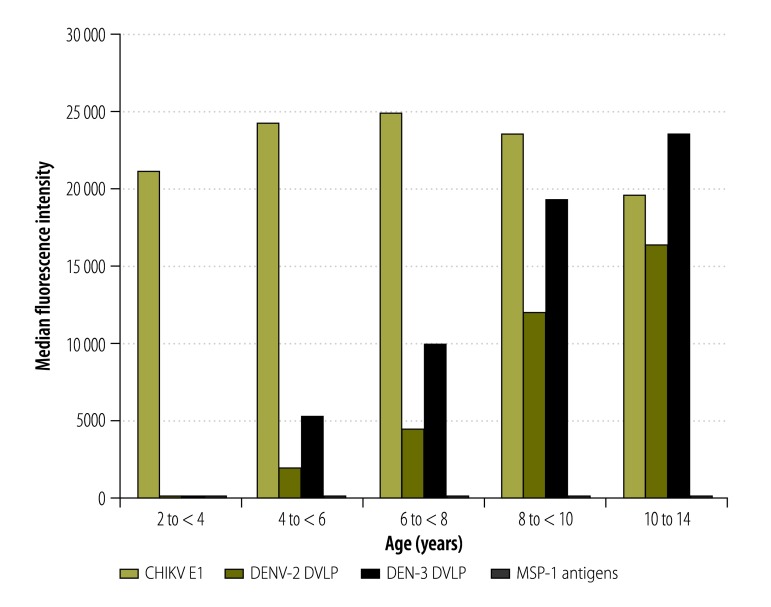
Age-specific fluorescence intensities recorded in multiplex bead assays based on antigens representing chikungunya and dengue viruses and *Plasmodium falciparum*, Haiti, 2014

**Fig. 4:**
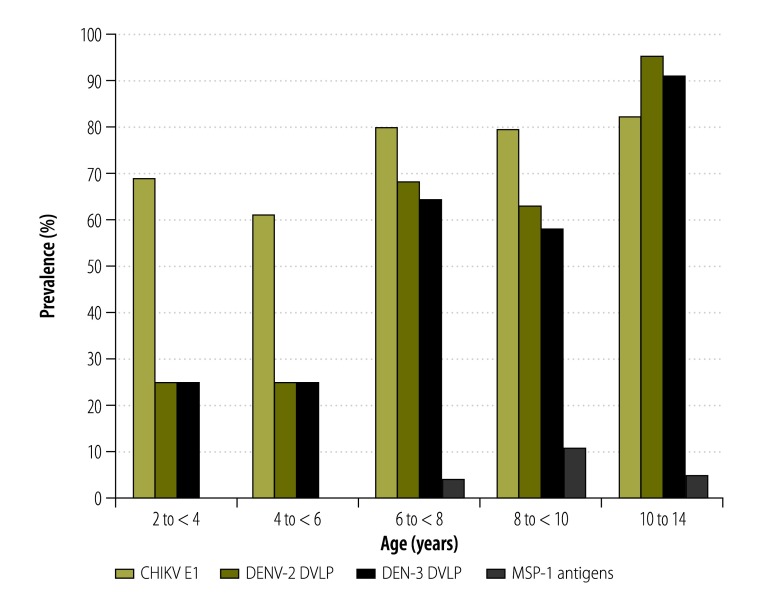
Age-specific prevalence of exposure to chikungunya and dengue viruses and *Plasmodium falciparum*, Haiti, 2014

In the assays based on dengue virus antigens, median intensities (Fig. 3) and prevalence (Fig. 4) increased with the age of the child. In the assays based on chikungunya virus antigen or malarial antigens, however, there was no indication that age had any effect on median intensities (Fig. 3) or prevalence (Fig. 4).

CHIKV E1: chikungunya virus envelope 1; DVLP: dengue virus-like particle; MSP-1: merozoite surface protein 1.

Notes: The assays were used, with six test antigens, to investigate blood spots from a cross-sectional sample of 127 children. The fluorescence intensities shown are each the result of subtracting background fluorescence, from wells containing no primary antibody, from the mean of the median fluorescence intensities of duplicate wells. As the three *P. falciparum* antigens tested – i.e. 19-kDa, 42-kDa and 42-kDa fractions of the MSP-1 clones 3D7, 3D7 and FVO, respectively – gave almost identical results, they are not shown separately.

CHIKV E1: chikungunya virus envelope 1; DVLP: dengue virus-like particle; MSP-1: merozoite surface protein 1.

Notes: Multiplex bead assays were used to investigate blood spots, from a cross-sectional sample of 127 children, for immunoglobulin G responses to six antigens representing the three pathogens.

### Correlations

#### Inter-pathogen

The Spearman’s correlations indicated no significant cross-reactivity among our three study pathogens – i.e. between chikungunya virus and either dengue virus (*r*^2^ < 0.08; *P* > 0.12) or *P. falciparum* (*r*^2^ < −0.1; *P* > 0.5) or between dengue virus and *P. falciparum* (*r*^2^ < 0.09; *P* > 0.08).

#### Inter-antigen

In contrast, in terms of positivity in the bead assays, a strong correlation existed among the three malaria antigens (*r*^2^ > 0.59; *P* < 0.001) and between the two dengue antigens (*r*^2^ = 0.96; *P* < 0.001).

### Spatial analysis

**Fig. 5:**
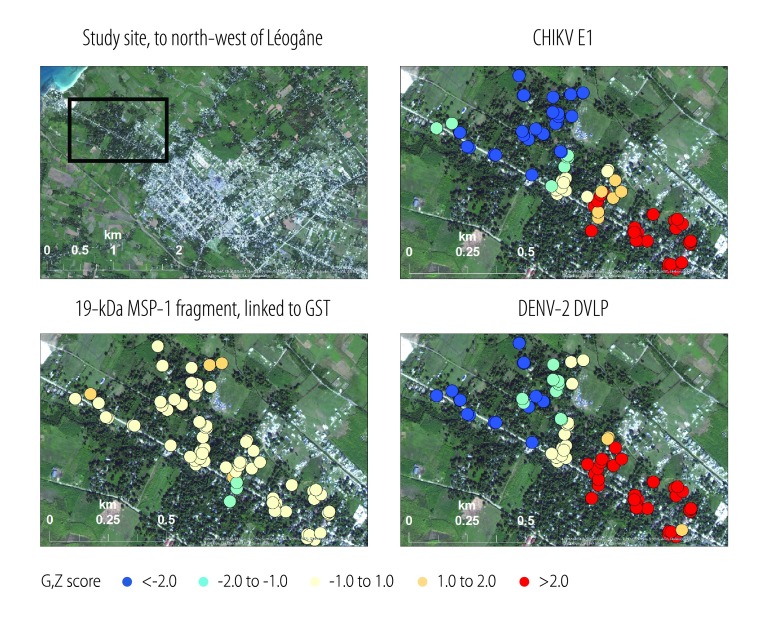
Spatial analysis of fluorescence intensities recorded in multiplex bead assays based on antigens representing chikungunya and dengue viruses and *Plasmodium falciparum,* Haiti, 2014

Clusters identified in the spatial analysis of the study area, which lies immediately outside urban Léogâne, are shown in Fig. 5 (available at: http://www.who.int/bulletin/volumes/94/11/16-173252). In general, the detected IgG responses to the chikungunya virus antigen and DENV-2 virus-like particle appeared to get weaker the further north-west the sampling site – i.e. the further away from the city of Léogâne and the closer to the ocean. Conversely, the corresponding responses to the 19-kDa malarial antigen linked to glutathione-*S*-transferase exhibited no discernible spatial pattern – although they were, in general, relatively weak.

CHIKV E1: chikungunya virus envelope 1; DVLP: dengue virus-like particle; GST: glutathione-*S*-transferase; MSP-1: merozoite surface protein 1.

Notes: The assays were used, with three test antigens, to investigate blood spots from a cross-sectional sample of 127 children. In the spatial analysis of the data, Z scores for the Getis–Ord *G*_i_ statistic were aggregated and displaced from their actual locations. Blue and red circles indicate, respectively, so called low spots and high spots – i.e. clusters where children had immunoglobulin G responses to the test antigen that were significantly lower and higher, respectively, than the mean value.

## Discussion

The results from the bead assay indicate that infection with chikungunya virus only became common in Haiti in 2014 and that there was rapid transmission of the virus after its introduction into an immunologically naive Haitian population. By the first week of 2015, there were 64 695 suspected cases of chikungunya virus infection in Haiti and – since there were over 500 000 cases reported, at the same time, in the neighbouring Dominican Republic – even this high number is likely to have been an underestimate because of underreporting.^13^ A nationwide household survey conducted by the Igarapé Institute indicated that 9.2% of Haitians had been infected with the virus by May 2014 – meaning there had been almost one million cases of infection within a month of the introduction of the virus into Haiti.^37^ Such an explosive outbreak was also indicated by our bead assay results, which indicated that the prevalence of exposure to chikungunya virus among children in the town of Ca Ira increased from 0% (0/61) in 2013 to 76.6% – i.e. 144 of the 188 children in the combined longitudinal cohort and cross-sectional sample – in 2014.

Our bead assay was sensitive enough to show both an initial immune response and an anamnestic response to the DENV-2 and DENV-3 virus-like-particles and it appeared to be unaffected by cross-reactivity among the three pathogens we investigated. It also indicated an increasing gradient, in the level of IgG responses to chikungunya virus and dengue virus antigens, running from an urban area towards the beach. One reason for this gradient may be the preference of *Ae. aegypti* and *Ae. albopictus* for habitats with relatively lower salinity and/or wind activity.^38^ The relatively high density of the human population in the more urban areas we investigated may also favour transmission.

Our results relating to dengue virus were consistent with those of previous studies on dengue in Haiti. Prior to 1969, dengue virus had not been reported in Haiti. In a study conducted in Port-au-Prince between 1969 and 1971, however, antibody responses to this pathogen were detected in 43%, 60% and 76% of the subjects aged 1–5, 6–10 and 41–50 years, respectively.^9^ In 1996, in a later study in Port-au-Prince, 85% of the children aged 6–13 years who were screened with a neutralizing antibody test were found positive for at least one dengue virus serotype.^39^

Our results relating to chikungunya virus, although based on a relatively small sample, are consistent with those of an unpublished but much larger study conducted in early 2015. The subjects of this larger study, which was focused on malaria, were checked for IgG responses to the chikungunya virus we used in the bead assay. The seroprevalence of such responses was found to be 82% in urban areas throughout Haiti – similar to the 79% found in our urban study site – and only 45% in rural areas (Eric Rogier, CDC, Atlanta, USA, personal communication, February 2016). In Haiti, rural areas are generally at higher altitudes than urban areas and the cool temperatures at high altitudes tend to limit vector densities.

Multiplex bead assays have been found to be at least as sensitive as ELISA^40^^–^^42^ and are relatively efficient since they require only 125 nL of specimen per well, in a 96-well format, while accommodating up to 100 antigens or data points per well. They can deliver efficiencies of cost, sample and labour while providing the opportunity to monitor the prevalence of exposure to multiple pathogens simultaneously. By analysing fluctuations in IgG responses to several antigens over time, with multiple blood samples from the same individual, such assays could also define exposure histories. Given the confirmed autochthonous transmission of Zika virus in Haiti and across the Americas,^20^^,^^43^ exposure histories are urgently needed. At a time when funding for the surveillance of a single disease is becoming increasingly difficult to defend, the versatility offered by multiplex bead assays could permit the longitudinal and cost–effective monitoring of multiple pathogens within a single study.
